# The interplay between Mn and Fe in *Deinococcus radiodurans* triggers cellular protection during paraquat-induced oxidative stress

**DOI:** 10.1038/s41598-019-53140-2

**Published:** 2019-11-20

**Authors:** Sandra P. Santos, Yang Yang, Margarida T. G. Rosa, Mafalda A. A. Rodrigues, Claire Bouthier De La Tour, Suzanne Sommer, Miguel Teixeira, Maria A. Carrondo, Peter Cloetens, Isabel A. Abreu, Célia V. Romão

**Affiliations:** 1 ITQB NOVA, Instituto de Tecnologia Química e Biológica António Xavier, Universidade Nova de Lisboa, Av. da República, 2780-157 Oeiras, Portugal; 2 Present Address: iBET, Instituto de Biologia Experimental e Tecnológica, Apartado 12, 2781-901 Oeiras, Portugal; 3 ESRF- The European Synchrotron, CS40220, 38043 Grenoble, Cedex 9 France; 4 Institute for Integrative Biology of the Cell (I2BC), CEA, CNRS, Univ. Paris-Sud, Université Paris-Saclay, 91198 Gif sur Yvette, France

**Keywords:** Chemical biology, Microbiology

## Abstract

The bacterium *Deinococcus radiodurans* is highly resistant to several stress conditions, such as radiation. According to several reports, manganese plays a crucial role in stress protection, and a high Mn/Fe ratio is essential in this process. However, mobilization of manganese and iron, and the role of DNA-binding-proteins-under-starved-conditions during oxidative-stress remained open questions. We used synchrotron-based X-ray fluorescence imaging at nano-resolution to follow element-relocalization upon stress, and its dependency on the presence of Dps proteins, using *dps* knockout mutants. We show that manganese, calcium, and phosphorus are mobilized from rich-element regions that resemble electron-dense granules towards the cytosol and the cellular membrane, in a Dps-dependent way. Moreover, iron delocalizes from the septum region to the cytoplasm affecting cell division, specifically in the septum formation. These mechanisms are orchestrated by Dps1 and Dps2, which play a crucial role in metal homeostasis, and are associated with the *D. radiodurans* tolerance against reactive oxygen species.

## Introduction

The bacterium *Deinococcus (D*.) *radiodurans*, is a highly resistant organism to several conditions, namely radiation, desiccation, or oxidation. The complex mechanisms behind the resistance to such stresses remain unclear, although they have been the focus of several studies^1,2^. What is proposed is that *D. radiodurans* avoids cell death by protecting its proteome against oxidative stress^3,4^, using a high intracellular Mn/Fe ratio^5,6^. Manganese protective role is not only associated with an enzymatic system (*e.g*., as a cofactor of Mn-superoxide dismutase, MnSOD) but also with a non-enzymatic system relying on the accumulation of small Mn^2+^-antioxidant complexes^7,8^. Manganese forms complexes with small molecules, such as orthophosphate, and pyrophosphate, and these can react with reactive oxygen species (ROS) formed under stress conditions^9–11^.

Although neither the homeostasis of manganese nor the intracellular localization and formation of the Mn^2+^-complexes are yet fully understood, *D. radiodurans* high cellular manganese concentration was associated with the existence of electron-dense granules located at the center of its nucleoids^4^. These electron-dense granules are also known as phosphate granules and were reported in *D. radiodurans* by several publications [e.g.^1,12,13^]. The exact composition of these granules is not known, but similar structures in other organisms were reported to contain a high amount of phosphate in the form of polyphosphate, pyrophosphate, and orthophosphate^14–16^, and also other ions, such as calcium, magnesium, and potassium^14,17,18^.

*D. radiodurans* does not have any gene that encodes for a ferritin-like protein but it expresses two DNA-binding proteins under starved conditions: Dps1 (DR2263) and Dps2 (DRB0092); *in vitro*, both can store manganese and iron and have the ability to bind/protect DNA^19–21^. Dps1 shows distinct oligomeric forms depending on cellular growth phase and additionally can change its oligomeric form in response to the addition of manganese and iron^21^. Dps2 was proposed to have a role in Fe-storage since in *in vitro* conditions it has a higher iron storage capacity (1.6×) than Dps1, moreover the electrostatic potential surface of the internal cavity of Dps2 is more negative than Dps1 and thus more prone to store iron^21^. Adding to this, its non-cytoplasmic localization and the fact that iron localizes in the septum region, suggests that Dps2 can be involved in Fe-sequestering^4,20^. Recently we showed that *D. radiodurans* has two dodecameric forms of Dps2, one associated with the membrane (Dps2_M_) and another present in the cytoplasm (Dps2_C_)^22^, suggesting that Dps2 may be involved in Fe-trafficking.

Taking into account the above open questions, we asked if manganese and iron distributions are important for *D. radiodurans* oxidative stress response and if its two Dps are involved in the overall process. To correlate the protection mechanisms of *D. radiodurans* with the presence of manganese, iron, and both Dps, we studied the wild-type and *dps* knockout mutants and followed element localization by state-of-the-art synchrotron X-ray fluorescence nano-imaging (nano-XRF). This technique allows precise intracellular localization of trace elements within the cellular context. Obtained results were contextualized with the cellular localization of GFP-tagged Dps proteins as determined by epifluorescence microscopy.

Our results show that cell protection against ROS depends not only on a proper Mn/Fe ratio but maybe more importantly, on the correct cellular localization and trafficking of the metals. The *Dr*Dps-dependent re-distribution of these ions contributes to the intracellular protection against oxidative stress of the radiation-resistant bacterium *D. radiodurans*.

## Results and Discussion

### Mn and Fe at the forefront of oxidative stress management

**Figure 1:**
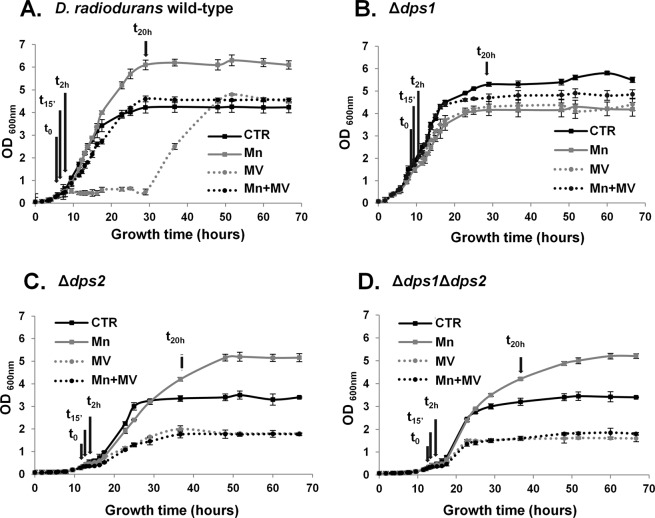
*D. radiodurans* cells subjected to different conditions. Growth curves of *D. radiodurans* cells strains: (**A**) wild-type, (**B**) Δ*dps1*, (**C**) Δ*dps2*, (**D**) Δ*dps1*Δ*dps2*, grown in M53 medium with the addition at time 0 (OD = 0.3) of water (Control condition, black line), manganese (Mn, gray line), methyl viologen (MV, dotted gray line), and manganese followed by the addition of methyl viologen (Mn + MV, dotted black line). The time points analyzed are represented by the arrows and correspond to times 0, 15′ (early exponential phase), 2 h (mid-exponential phase), and 20 h (stationary phase). Data presented are from four biological replicates (+/−sd).

To evaluate the protective effect of manganese in *D. radiodurans* when subjected to oxidative stress, we used methyl viologen (MV, paraquat) at early-exponential growth to induce superoxide production, in the presence or absence of added manganese (Fig. 1A, Supplemental Fig. 1).

After the oxidative stress stimulus, cells immediately stopped their normal growth but could recover after 30 hours (Fig. 1A). The addition of manganese before MV (Mn + MV) protected the cells against oxidative stress (Fig. 1A). Interestingly, Δ*dps1* strain does not feel the stress as wild-type (WT) cells do (Fig. 1B). This strain showed no growth arrest after the MV addition, and consequently, the pre-treatment with manganese had no visible effect. In fact, Δ*dps1* cells phenocopy the Mn + MV condition in WT cells (Fig. 1B). *D. radiodurans* mutant strains Δ*dps2* and Δ*dps1*Δ*dps2* (Fig. 1C,D) show similar growth profiles under control and MV conditions, suggesting that the absence of Dps2 is sufficient to induce the observed phenotypes. Cellular growth profiles show that the cells are not able to recover to the same growth level as WT do after oxidative stress, even in the presence of extra manganese. Still, Δ*dps2* and Δ*dps1*Δ*dps2* regain the ability to grow to higher optical densities in the presence of manganese alone, a trend observed in the WT strain but not in Δ*dps1* (Fig. 1B–D). The observed effect of higher optical densities in the presence of Mn has been previously reported for the *D. radiodurans* WT strain (e.g.^23,24^).

**Figure 2:**
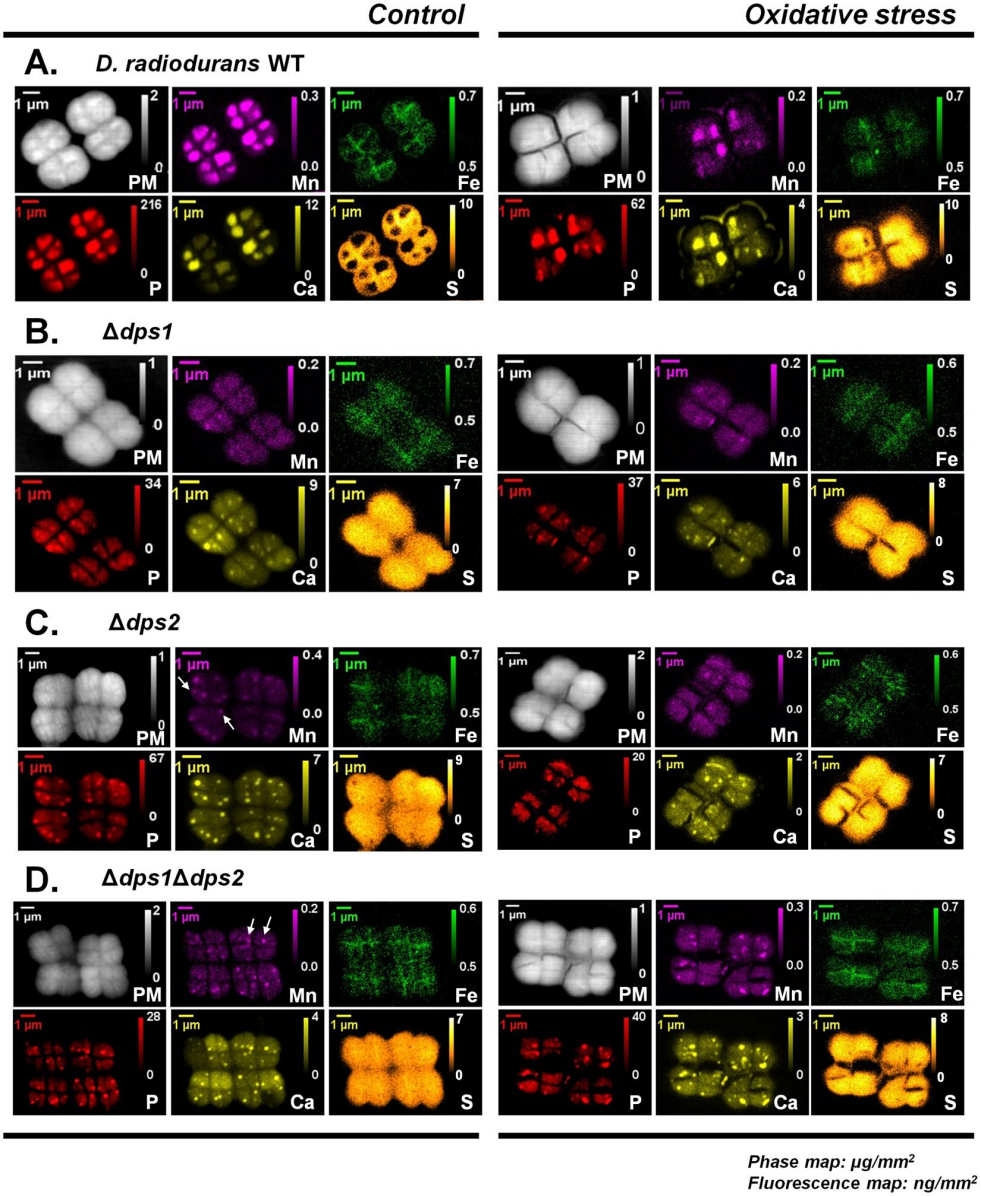
Localization of elements in *D. radiodurans* cells using X-ray fluorescence nano-imaging (nano-XRF). Elements mapping in *D. radiodurans* (**A**) wild-type, *(***B***)* Δ*dps1, (***C**) Δ*dps2* and (**D**) Δ*dps1*Δ*dps2*. The samples analyzed were in control and oxidative stress conditions at time point 2 hours. Oxidative stress was promoted by the addition of methyl viologen (MV). Elemental areal density quantification bar (ng/mm^2^) for each element is presented. In panel (C,D) in control conditions, the small regions where Mn is concentrated are marked with white arrows. Data presented are from three biological replicates (+/−sd). Each condition was measured by nano-XRF at least in 12 cell tetrads.

**Figure 3:**
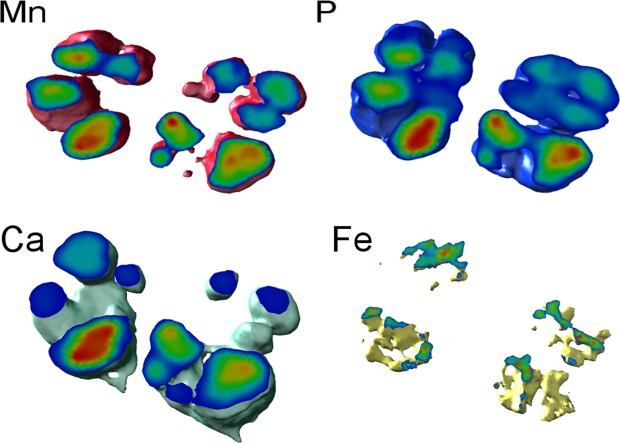
3D localization of the elements in *Deinococcus radiodurans* using X-ray fluorescence nano-imaging (nano-XRF). 3D elemental imaging in *Deinococcus radiodurans* wild-type cells under control conditions, the elements present are manganese, phosphorous, calcium and iron.

To assess metal distribution during the cellular response to oxidative stress, we followed metal-intracellular localization using nano-XRF. Manganese, phosphorus, and calcium, in wild-type *D. radiodurans*, are co-localized in rich elemental cellular regions that resemble electron-dense granules, while iron is mostly localized in close proximity to or within the septum membrane (Figs. 2A, 3).

When an oxidative stress stimulus occurs, manganese is mobilized to the cytoplasm and is transported to the outer region of the membrane (Fig. 2A, Supplemental Fig. 2), while the iron is mostly dispersed throughout the cell (Fig. 2A). It is possible that the mobilization of manganese ultimately results in membrane protection, which would agree with the protective effect exerted by the external addition of manganese before MV addition, as shown above. This result corroborates previous studies, in which the addition of manganese before the exposure to radiation protects the cells^5^. Notably, phosphorous and calcium follow manganese redistribution (Fig. 2A, Supplemental Fig. 2). It has been reported that manganese can form complexes with phosphate molecules (Pi) that can detoxify ROS, probably in a Mn^2+^-Pi form^10,25^. Analysis of *D. radiodurans* WT cells after being subjected to the addition of manganese alone showed that the regions rich is several elements, which we proposed to be electron-dense granules, tend to increase in size (Supplemental Fig. 3A), and exhibit higher amounts of Mn, P and Ca, when compared to control condition (Fig. 2, Supplemental Figs. 3, 4). So the formation of Mn^2+^-Pi complexes may not be the sole form of Mn-complexes in *D. radiodurans*.

In all *D. radiodurans dps* mutant strains, the intracellular amount of Mn is similar to that of WT, while P amounts decrease (Supplemental Fig. 4). The ability that the mutant strains Δ*dps1*, Δ*dps2*, and Δ*dps1*Δ*dps2*, have to form putative electron-dense granules is compromised since manganese and phosphorus are homogeneously distributed in the cytoplasm. However, small regions where these metals are concentrated are still visible in the cells of Δ*dps2* and Δ*dps1*Δ*dps2* (Fig. 2C, D- white arrows). At this point, it is not possible to assess if the nature of these regions is the same as that of the WT electron-dense granules or if they constitute another type of element-containing bodies. Furthermore, nano-XRF results show that iron is concentrated in the septum region in WT cells, while it looks more widespread throughout the cell in all knockouts mutants (Fig. 2, Supplemental Figs. 4, 5). It is important to notice that the Mn/Fe is *ca*. 0.8 ± 0.15 for the conditions tested and in all *D.radiodurans* strains, except for when external manganese was added to the cell growth media (Supplemental Fig. 3).

Therefore, we can say that the resistance and recovery capabilities of *D. radiodurans* to oxidative stress and the intracellular distribution of elements are dependent on the presence of Dps1 and Dps2. The absence of Dps1 renders the cells insensitive to oxidative stress as they do not interrupt their normal growth when MV is added as WT cells do (Fig. 1A,B). On the contrary, the absence of Dps2 (Δ*dps2* and Δ*dps1*Δ*dps2*) is sufficient to compromise cell growth and the ability of the cells to recover from oxidative stress or to use Mn as a protecting agent (Fig. 1C,D). Thus we next asked what the role of both proteins in this process is.

### Different Dps protein forms determine their cellular localization

**Figure 4:**
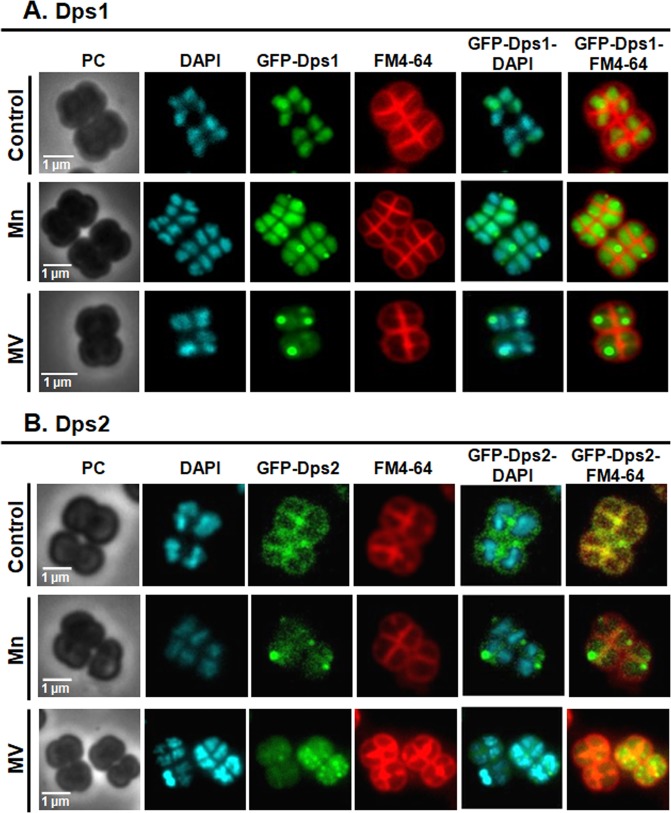
*D. radiodurans* GFP*-*Dps1 and GFP*-*Dps2 cellular localization using epifluorescence microscopy. The samples analyzed were *D. radiodurans* cells under control, manganese (Mn) and methyl viologen (MV) conditions at 2 hours. Cell images are represented in phase contrast (PC), stained for DNA using DAPI (blue), fluorescence for GFP-Dps constructs (green), and the membranes stained with FM4–64 (red). The two last columns correspond to the overlay of GFP-Dps with DAPI and GFP-Dps with FM4–64. Data presented are from four biological replicates (+/−sd).

**Figure 5:**
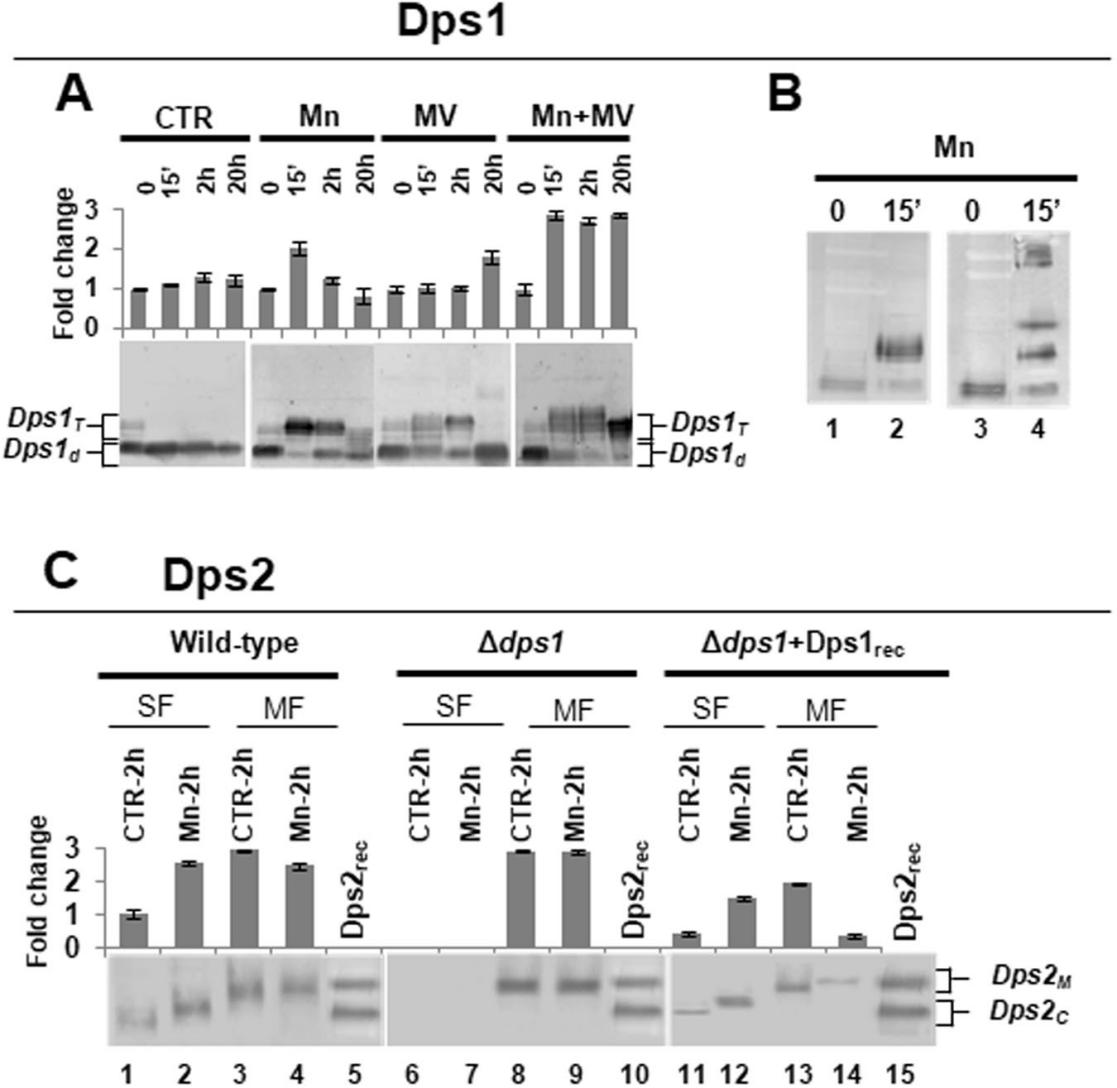
Detection of Dps1 and Dps2 in *D. radiodurans* cellular extracts. (**A**) Dps1 detection in the following conditions: control (CTR), manganese (Mn), methyl viologen (MV) and manganese followed by the addition of methyl viologen (Mn + MV). Dps1_d_ corresponds to the dimeric form of Dps1 which is associated with the DNA (Dps1_DNA_) and Dps1_T_ to the trimeric form (Dps1_T_) which is phosphorylated (Dps1_P_). The time points analyzed correspond to 0, 15’ (early exponential phase), 2 h (mid-exponential phase), and 20 h (stationary phase). (**B**) Detection of phosphorylation in Dps1 from *D. radiodurans* wild-type cellular extracts, subjected to manganese condition at time 0 and 15’, without (lanes 1, 2) and with (lanes 3, 4) Phos-tag AAL-107. (**C**) Detection of Dps2 in soluble and membrane fractions in *D. radiodurans* wild-type, Δ*dps1* strains and after addition of Dps1 recombinant pure protein (Dps1_rec_) to the cellular extract of Δ*dps1*. The soluble (SF, lanes 1, 2, 6, 7, 11 and 12) and membrane (MF, lane 3, 4, 8, 9, 13 and 14) fractions were isolated from control (CTR) and manganese (Mn) conditions at time 2 h. Dps2 recombinant (Dps2_rec_ - lanes  6-9 and 11-14), in which Dps2_M_ corresponds to the Dps2 dodecamer (279 kDa), and Dps2_C_ corresponds to Dps2 dodecamer without N-terminal tails (232 kDa). (**A,C**) Bar charts represent the quantification of the total Dps amount using ImageJ. Data presented in panel *A* are from four biological replicates (+/−sd), and in panel *B* and *C* are from two biological replicates (+/−sd). (A.B) Correspond to the full-length blots; (**C**) full-length blots are presented in Fig. S8A.

Although Dps are known as dodecameric proteins, we previously have shown that Dps1 can be dimeric, trimeric or dodecameric according to the condition that it was subjected^21^. Based on our previous data the dimeric Dps1 is associated with DNA^21^. Here, we show that under control conditions this association is observed during the exponential growth phase of the bacteria since GFP-Dps1 fluorescence signal superimposes with the DAPI staining of DNA (Fig. 4, Supplemental Fig. 6) and that, during this growth phase, Dps1 remains mostly dimeric (Fig. 5A)^21^. Immediately after Mn or MV addition, Dps1 changes its oligomeric form from a dimer to a trimer (Fig. 5A) as also shown previously^21^, and the protein appears dispersed throughout the cell with a stronger signal in specific regions that are consistent with the electron-dense granules (Fig. 4A). This change in oligomerization state is concomitant with the phosphorylation of the protein in, at least, five different residues (Fig. 5B), but does not depend on it^21^. The phosphorylation sites of Dps1 are not known, but interestingly, Dps1 has a putative phospho-motif (D_145_AR**T**_**148**_QVADLV) that can be targeted by the radiation pyrroloquinoline quinone (PQQ) inducible protein kinase (RqkA) (DR2518)^26^.

Our previous work shows that Dps2 is a stable dodecamer and it can store iron or manganese, but when is in the presence of equal amounts of iron and manganese, the protein is selective towards iron, incorporating approximately double amounts of iron than of manganese^21^. It is interesting to note that in WT cells grown in control conditions, the localization of Dps2 coincides with the iron distribution, mostly at the septum region (Figs. 2A, 4B). Consistently, Dps2 was primarily detected in the membrane fraction of these cells (Fig. 5C, Supplemental Fig. 8A). Upon MV addition, Dps2 and iron became homogeneously distributed throughout the cells; Mn appeared to leave the electron-dense granules and also became widespread all over the cell, and appeared in the outer part of the plasma membrane (Fig. 2A, Supplemental Fig. 2). Upon addition of external Mn, Dps2 was no longer localized in the setptum membrane, instead was localized in the cytosol (Figs. 4B, 5C, Supplemental Fig. 8A). Thus, we propose that *in vivo* conditions in the presence of an excess of manganese, Dps2 can store manganese metal as well as iron. Also, it is interesting to note that the Dps2 crystal structure previously determined, showed a metal iron site coordinated by D_132_(X)D_133_(X_60_)D_193_(X)*N*_195_(X_5_)I_200_^27^. This metal site is close to the putative Dps2 Mn-binding motif, N(X_3_)D^28^. However, from the crystal structure, the binding motif includes N(X_5_)I instead of N(X_3_)D, in which the coordination of I_200_ is through the oxygen from the main chain. Thus it is plausible that Dps2 will manage an increase in cytoplasmic manganese occurring with the external addition of this metal or as a result of oxidative stress, acting then as a manganese transporter in the cell. In this sense, both iron and manganese mobilization would be mediated by Dps2. The absence of Dps2 results in the disruption of the WT patterns of distribution of both metals (Figs. 2, 3).

Phosphorous and calcium distributions are also affected in Δ*dps2* and follow the manganese distribution pattern. While the formation of Mn^2+^-Pi complexes^7,8^ can explain phosphorous co-distribution with manganese, the calcium profile was unexpected. If calcium distribution follows the manganese one, which depends on Dps2, then Dps2 should also influence calcium localization. Indeed, Dps2 can bind Ca^2+^ ^29^, which further supports this hypothesis.

### A direct functional link between Dps1 and Dps2

Remarkably, the detachment of Dps2 from the membrane is dependent on the presence of Dps1, since in Δ*dps1* cellular extracts all Dps2 is membrane-associated, and this can be reversed by the addition of recombinant Dps1 (Fig. 5C- lanes  6-9, 11-14 Supplemental Fig. 8A). However, Dps1 is not directly responsible for Dps2 N-terminal cleavage, since adding recombinant Dps1, in the trimeric form (Dps1_T_), to the full-length dodecameric form of recombinant Dps2 (Dps2_M_) does not lead to a change in Dps2 molecular mass (Supplemental Fig. 7). Also, Dps1 phosphorylation may have a role in this process since the trimeric form of Dps1 detected in *D. radiodurans* cellular extracts is always phosphorylated.

The direct consequence of the above mechanism is that, when Dps1 is not present, Dps2 cannot leave the membrane, and if Dps2 is shuttling metals, such as iron and manganese, their homeostasis will be affected. Accordingly, the absence of Dps1 is sufficient to destabilize the formation or growth of the electron-dense granules, where manganese, phosphorus, and calcium are concentrated, as our data indicates (Fig. 2B). In the initial phase of WT growth (time 0), a small proportion of the Dps1 trimeric form is detected (Fig. 5A), which may be important to initiate the formation of the granules, through the action of cytoplasmic Dps2 (Dps2_C_). Thus, in Δ*dps1*, these regions are much smaller than in the WT cells (Fig. 2A,B).

While Dps1 affects Dps2 localization, the absence of Dps2 does not affect the oligomerization states of Dps1 (Supplemental Fig. 8B). However, in all the three *dps* knockout mutants, the correct distribution of iron and manganese to the septum and granules, respectively, is abolished. According to our model, the absence of MV-sensitivity by Δ*dps1* cells may be due to the exclusive presence of Dps2_M_ that results in widespread distribution of manganese, which would protect the cells against oxidative stress, as the addition of external manganese does in WT cells.

In summary, Dps1 and Dps2 appear to act in a concerted way in *D. radiodurans* to regulate manganese and iron homeostasis and act in the formation of the regions rich in elements such as manganese and phosphorus that we propose to correspond to electron-dense granules.

### Dps–dependent iron localization is essential for septation

Under control conditions, cell division occurs as an orderly process, where tetrads are generated by the formation of two septa in opposite sides perpendicularly to the cell wall, forming two cells^1,30^. Moreover, manganese is concentrated in the rich elemental regions, whereas iron is localized in the membrane septum (Fig. 2A). Interestingly, all the *dps* knockout mutants show a strong phenotype in cell division, as cells seem to have difficulty in performing septation to form the new tetrads (Supplemental Fig. 9). We hypothesize that the presence of Dps2 in the membranes of *D. radiodurans* may play a role in cell septation since its tendency to distribute to the cytoplasm correlates with the severity of the phenotype. Both Δ*dps2* and Δ*dps1*Δ*dps2* mutants have the most pronounced phenotype. Their cells have irregular forms, and the septum formation seems to be affected. This is visible even under control conditions (Supplemental Fig. 9). Accordingly, both WT and Δ*dps1* strains have similar doubling times of *ca*. 2 hours, while Δ*dps2* and Δ*dps1*Δ*dps2* grow slower with a doubling time of *ca*. twice that of WT and Δ*dps1* (Supplemental Fig. 10). Even though the most striking difference amongst WT and the *dps* knockout mutants strains is that the mutants cells tend to aggregate (Supplemental Fig. 9), they still show a tetrad-like organization in the Δ*dps1* mutant cells, which is gradually lost in Δ*dps2* and more evidently in Δ*dps1*Δ*dps2*. These phenotypes can merely reflect the cells’ inability to accurately form the septum when Dps2 is not present or fully functional (in Δ*dps1*, Dps2 cannot exit the membrane). Thus, in both Δ*dps2* and Δ*dps1*Δ*dps2*, cellular division occurs in a disordered manner even under control conditions. This effect is much more pronounced when cells are subjected to a stress condition, where the septum region is profoundly damaged (Fig. 2C,D, Supplemental Fig. 9). This can explain why in both Δ*dps2* mutants the cells never recover after being subjected to MV promoted stress condition, even when pre-treated with manganese (Fig. 1C,D). This effect is reflected in the lag-phase of the different strains (Supplemental Fig. 10). Also, both Δ*dps2* mutants have longer lag-phases when compared with the WT cells, while the Δ*dps1* mutant has a slightly shorter lag-phase. It is interesting to observe that the cell septation phenotype observed in the Δ*dps2* mutants is similar to the one seen in the autolysin knockout mutant from murein hydrolase of *Staphylococcus aureus*, (AtlA)^31^. Although autolysins from *D. radiodurans* have not been studied, there are at least four genes predicted for N-acetyl muramoyl-L-alanine amidases, namely DR2394, DR1387, DR2567, and DR1632. The role of iron in the cell separation process remains unclear. Nevertheless, we cannot rule out that the effect observed in the cell division is related to the damage of DNA since Dps2 protects DNA under *in vitro* conditions^21^. If that is the case, the inhibition of the septum formation due to DNA damage could resemble the SOS system from *Escherichia coli* (e.g.^32^).

In summary, these data indicate that there is a link between Dps function, the presence of iron in the septum region, and cell septation. The association of Dps with the cell division process is only very poorly studied, although DpsA from *Streptomyces coelicolor* was proposed to be involved in the condensation of DNA to ensure an appropriate degree of nucleoid compaction during cell division^33^. Nevertheless, the question remains of whether Dps in *D. radiodurans* are also involved in the DNA compaction before cell division, DNA protection in addition to controlling Fe localization in the septum region.

### Electron-dense granules are multi-ion stores involved in stress response

Our data show the presence of rich elemental regions with high concentrations of manganese, phosphorous, and calcium that we propose to correspond to the electron-dense granules in *D. radiodurans*. Other elements, like potassium or copper, can also be found in these regions, but to a lesser extent (Supplemental Fig. 3). This is in agreement with previous work, in which under control conditions manganese is mostly not associated with proteins^8,10^. After a stress stimulus, manganese, phosphorus, and calcium are dispersed throughout the cell and into the membrane structures (Fig. 2A, Supplemental Fig. 2). As manganese and phosphorus migrate together, it is tempting to speculate that they probably exist in the form of Mn^2+^-Pi complexes and that these are directed to the membrane to play a role in the protection of macromolecules against oxidation, as previously proposed^9,10^. Noteworthy, no data is supporting the *in vivo* formation of Mn^2+^-Pi complexes in *D. radiodurans*, but manganese can form complexes with pyrophosphate or orthophosphate, in other organisms^9,34,35^. Also, *D. radiodurans* has enzymes, *e.g*., polyphosphate kinases (DR1939, DR0132) and exopolyphosphatase (DRA0185), capable of hydrolyzing polyphosphate^2,36^. As shown above, calcium follows the same redistribution pattern as phosphorus and manganese. As in other organisms, calcium mobilization to the membranes may serve to initiate stress-responsive signaling pathways involving kinases and should be investigated in the future. Our data do not allow to discriminate between free Ca^2+^ and Ca^2+^-complexes, and thus, we cannot rule out that calcium phosphate complexes can also exist intracellularly as previously observed in mitochondria^37^.

The rich elemental regions are devoid of sulfur, while calcium, manganese, and phosphorus, are compartmentalized in specific regions within the cell (Fig. 2A, Supplemental Fig. 3). The fact that in the *dps* knockout mutants sulfur does not appear as excluded from these regions suggests that the observed structures in these mutants are different from those observed in the WT cells.

In sum, the data presented here show the presence of rich elemental regions in *D. radiodurans* that act as reservoirs of calcium, manganese, and phosphorus and can be mobilized upon stress.

### *Dr*Dps as central players in oxidative stress response

**Figure 6:**
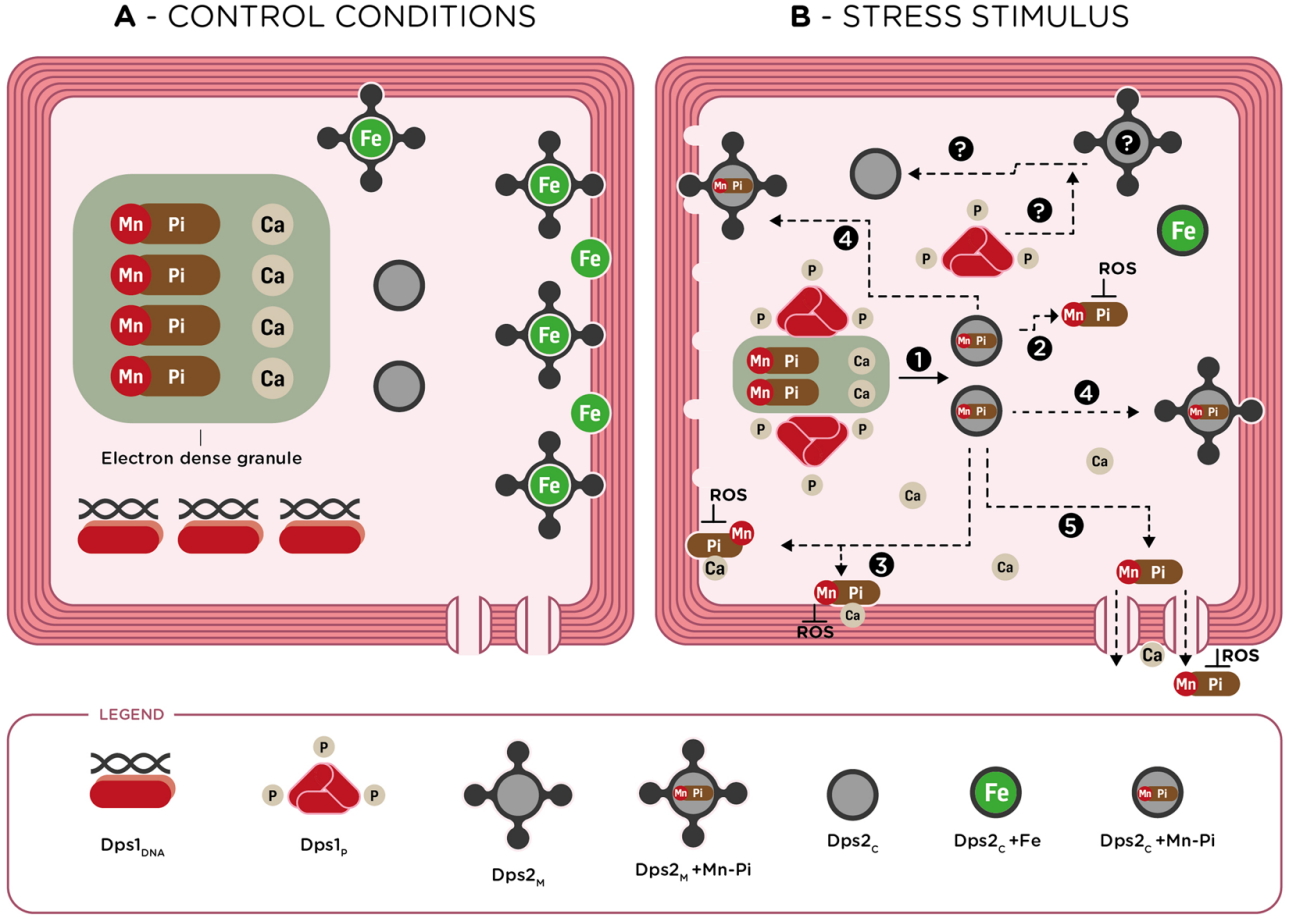
Proposed model for *D. radiodurans* protection mechanism after a stress stimulus, linking the cellular function of Dps1 and Dps2 with Mn and Fe homeostasis. (**A**) Under control conditions, Dps1 in its dimeric form is bound to DNA (Dps1_DNA_), and most of the Dps2 is attached to the membrane (Dps2_M_) through its N-terminal tail, but it is also present in the cytosol, without N-terminal tails (Dps2_C_). Phosphorous, probably phosphate (Pi), manganese (Mn) and calcium (Ca) are stored in the electron-dense granules that are highly rich in these elements. Iron (Fe) is mostly localized in the septum membrane region and can be stored by Dps2_M_. (**B**) Under oxidative stress, Mn, Pi, and Ca are released to the cytosol. Dps1 changes its oligomeric state from dimer to trimer, which is phosphorylated (Dps1_P_), and becomes mostly widespread in the cytoplasm with a higher concentration close to the electron-dense granules. A detachment of Dps2 from the membrane (Dps2_M_) and Mn and Pi release as Mn-Pi complexes to the cytosol (step 1) is dependent on Dps1_P_. Dps2_C_ is proposed to store Mn-Pi complexes and deliver them to the cytosol (Step 2), septum, and membrane regions (Step 3). Distribution of Mn-Pi complexes to the membrane and septum constitute a first protection barrier against ROS. Dps2_M_ and membrane Mn-transporters may be involved in the distribution of Mn-Pi throughout the membrane (Steps 4 and 5). Question marks represent open questions.

To summarize our findings, we propose a model (Fig. 6) showing the role of *Dr*Dps in manganese and iron homeostasis that contributes to the intracellular protection against oxidative stress of the radiation-resistant bacterium *Deinococcus radiodurans*. The model proposes that under control conditions this organism stores manganese in the rich elemental regions that we propose to be electron-dense granules together with other elements, namely phosphorus, and calcium, whereas iron is mostly localized in the septum region. Under these conditions, Dps1 associates with the DNA (^21^ and the data here presented), whereas Dps2 preferentially store/transport iron^21^, and localizes mostly at the septum region.

After being subjected to a stress stimulus, in our case oxidative stress promoted by MV addition, iron becomes dispersed throughout the cell, probably halting cell division. Manganese is also distributed throughout the cell, probably in the form of Mn^2+^ complexes with small molecules such as phosphate, by Dps2. Moreover, manganese appears in the outer part of the membrane, where it can protect membranes from further damage. Indeed, Mn-complexes have been proposed to play a crucial role in the scavenging of ROS upon stress conditions^9,10^. Calcium follows a similar distribution and may be responsible for the activation of stress-responsive signaling pathways involving kinase activation that may affect the phosphorylation state of Dps1.

The model now presented shows how Dps, including their different proteoforms, Dps1_DNA_/Dps1_P_, Dps2_M_/Dps2_C_, control the localization and stress-dependent relocalization of manganese together with other elements (phosphorous, calcium, and iron) (Fig. 6). Dps1 regulates processes such as the formation of the rich elemental regions and the detachment of Dps2 from the membrane, while Dps2_C_ plays a role as a metal transporter (Fe *vs*. Mn). The *Dr*Dps crosstalk and the interplay between iron and manganese are critical protection players in alleviating oxidative stress damage, in *D. radiodurans*. As the formation of superoxide anion and hydrogen peroxide are exceptionally rapid after irradiation^38^, we expect that the proposed model is also valid during *D. radiodurans* exposure to radiation, which ultimately leads to the formation of ROS.

## Material and Methods

### GFP-Dps1 and GFP-Dps2 bacterial strains and transformation

*D. radiodurans* strains expressing Dps proteins fused to GFP-tag were constructed by the tripartite ligation method^39^. Plasmid pFAP246 was the source for the cassette containing the GFP-tag and the resistance gene to chloramphenicol^40^. The gene sequence and the purity of the resulting tagged genes were verified by PCR and sequence analysis. Strains expressing tagged proteins were grown with aeration in TGY2x at 30 °C. Media were supplemented with chloramphenicol (3.5 µg/ml final concentration). *D. radiodurans* Δ*dps1*, Δ*dps2* and Δ*dps1*Δ*dps2* strains were previously described^41^.

### Deinococcus radiodurans growths

*D. radiodurans* cells wild-type, Δ*dps1*, Δ*dps2*, Δ*dps1*Δ*dps2*, GFP*-*Dps1 *and* GFP*-*Dps2 strains were grown in M53 medium (1.0% (w/v) casein peptone, 0.5% (w/v) yeast extract, 0.5% (w/v) glucose and 0.5% (w/v) NaCl) at 30 °C. Four independent growths were performed, and cells were collected at different time points. Methyl viologen (paraquat) is a compound that increases the intracellular production of superoxide anion and thus induces an oxidative stress condition^42^. Different concentrations of methyl viologen (MV) were initially tested from 0.5 mM to 5 mM, without and in the presence of 0.5 mM manganese, which was added 2 hours before or after MV addition (Supplemental Fig. S1). Based on these initial tests, the effect of different compounds was tested, namely: 0.5 mM manganese (II) (Mn) chloride, 1 mM MV and 0.5 mM manganese (II) chloride followed after 2 hours by the addition of 1 mM methyl viologen (Mn + MV). These compounds were added to the media at an optical density (OD_600nm_) of 0.3. A control (water addition) growth was performed and followed simultaneously. The different growths were obtained from bacterial inocula in later exponential phase and reliable growing conditions, important to achieve a reproducible lag time. Cells were collected at different time points: 0 (before adding any compound – OD = 0.3), 15′ (early exponential phase), 2 hours (mid-exponential phase), and 20 hours (stationary phase) after adding the different compounds. The viability of the cells was assessed using 10 μg/ml propidium-iodide dye. The cells were visualized on a Leica DM RA2 microscope.

### *Dr*Dps detection from soluble and membrane fractions

Cellular extracts from *D. radiodurans* wild-type in control conditions, and after manganese, methyl viologen and Mn + MV addition at time points 0, 15’, 2 h and 20 hours were ultracentrifuged at 200,000 × *g*, at 4 °C. Soluble and membrane fractions were quantified using Bradford (Bio-Rad)^43^ and modified Biuret methods^44^, respectively. A total of 30 µg of protein from each condition was loaded on a 12% PAGE, and the Dps1 protein bands were detected by Western-Blotting, as previously described^21^. Four biological replicates (+/−sd) were used.

Detection of Dps2 in soluble and membrane fractions in *D. radiodurans* wild-type, Δ*dps1* strains and after addition of Dps1 recombinant pure protein to the cellular extract of Δ*dps1* incubated during 1 h was also performed. The soluble and membrane fractions were isolated from control and manganese conditions at time 2 hours. Two biological replicates (+/−sd) were used.

### Mobility shift detection of phosphorylated proteins - Mn^2+^-Phos-tag Western blotting

The analyzed samples correspond to wild-type strain protein extracts, in manganese and control conditions, from time point 2 hours. Cellular extract containing 60 µg of total protein was centrifuged at 18,000 × *g*, 4 °C and the soluble fraction was injected on a 12% PAGE. Two gels were simultaneously run, without (control) and containing a 2 mM MnCl_2_ and 100 µM Phos-tag AAL-107 solution (Wako Chemicals USA, Inc.) in the resolving gel. These two solutions lead to the formation of a di-nuclear metal complex (1,3-bis[bis(pyridin-2-ylmethyl)amino]propan-2-olato di-manganese (II) complex - Mn^2+^-Phos-tag) acting as a selective phosphate-binding tag molecule^45^. Afterwards, the Dps1 protein bands were detected by Western-Blot. Two biological replicates (+/−sd) were used.

### Interaction between Dps1-Dps2

Both Dps (30 μg of recombinant Dps1 in the trimeric form and 30 μg of recombinant Dps2) were incubated for 1 hour at room temperature in 20 mM Tris-HCl pH 7.5 and 150 mM NaCl. Recombinant pure proteins were used as control. The different samples were loaded on a 12% PAGE.

### Dps1 and Dps2 cellular localization

*D. radiodurans* cells GFP-Dps1 and GFP-Dps2 strains in control, manganese and methyl viologen conditions were collected by centrifugation (11,000 × *g*, 1 min) at time points 0 (OD = 0.3) and 2 hours. Cells were resuspended in phosphate buffer saline (PBS), fixed with 3.7% paraformaldehyde for 15 min at room temperature followed by 30 min on ice, and stained with 2 µg/ml of 4,6-diamidino-2-phenylindole dihydrochloride (DAPI - Invitrogen) and 10 μg/ml of *N*-(3-Triethylammoniumpropyl)-4-(6-(4-(Diethylamino) Phenyl) Hexatrienyl) Pyridinium Dibromide (FM4–64 - Invitrogen). DAPI is a dye that stains nucleoid DNA with blue color (wavelengths of excitation/emission - 350/470 nm), and FM 4–64 is a dye that stains membranes with red color (excitation/emission − 515/640 nm). FITC filter (fluorescein-isothiocyanate-excitation/emission 495/517 nm) was used to visualize GFP-tag constructs. Cells were visualized on a Leica DM RA2 microscope, and the images were captured with a charge-coupled device (CCD) camera. The images were further processed using ImageJ software. Two biological replicates (+/−sd) were used.

### Synchrotron X-ray phase contrast and fluorescence nano-imaging

An aliquot of 1 ml from a growth of *D. radiodurans* cells wild type, Δ*dps1*, Δ*dps2* and Δ*dps1*Δ*dps2* strains in control and methyl viologen conditions were collected at time point 2 hours. Cells were fixed using 3.7% paraformaldehyde, then washed 2 times in PBS and stored frozen at −20 °C. Cells were afterwards resuspended in 25 µl of water, and then 1 µl was added onto the surface of a Si_3_N_4_ membrane (Silson Ltd) with the size of 1.5 mm × 1.5 mm and the thickness of 500 nm. Cells were air dried and then mounted on the cell support. Correlative imaging of both X-ray phase contrast and X-ray fluorescence (nano-XRF) was performed on the same sample to investigate its morphology and elemental content. Experiments were performed under vacuum (~1e^−7^ mbar) at room temperature on the Nano-Imaging beamline ID16A-NI of the European Synchrotron Radiation Facility (ESRF, Grenoble). The X-ray excitation energy was 17 keV, and all relevant elements were detected using their K-level emission lines. A multilayer coated fixed curvature Kirkpatrick-Baez (KB) focusing mirror system^46^ provides the nanofocus (~30 nm) and a very high flux of 4.1 × 10^11^ ph/s from the broad bandpass (1%) at 17 keV. X-ray phase contrast imaging was firstly performed by recording magnified Fresnel projection images with an equivalent pixel size of 15 nm. Quantitative phase maps were retrieved^47^ and converted to mass density (µg/mm^2^) in all figures. Nano-XRF measurements were performed subsequently with a step size of 40 nm or 50 nm and a dwell time of 50 ms^48^. The summed spectrum recorded with a 6-element silicon drift detector (Sensortech, UK) was fitted with the open source software PyMCA^49^. The absolute calibration to the elemental areal density (ng/mm^2^) was determined by a thin film standard (AXO Dresden GmbH). The average areal density’ corresponds to the mean inside the cells of the amount per unit surface of a given element. The total intracellular amount of an element corresponds to the integral of the areal density over the cell area (surface). The total amount is equal to the average areal density multiplied by the area covered by the cell. As the size of the cells does not vary significantly, both quantities are more or less equivalent. Three biological replicates (+/−sd) were used. Each condition was measured by nano-XRF at least in 12 cell tetrads.

### Significance statement

Re-distribution of intracellular Mn and Fe by Dps as key players for oxidative protection in *Deinococcus radiodurans*.

## Supplementary information

Supplementary Information
