# Supplementary material for: The interplay between Mn and Fe in Deinococcus radiodurans triggers cellular protection during paraquat-induced oxidative stress
Source: Sci Rep. 2019 Nov 20;9:17217. doi: 10.1038/s41598-019-53140-2 (PMC6868200; doi:10.1038/s41598-019-53140-2)
Supplement: Supplementary file 1 — Supplementary Information [file 41598_2019_53140_MOESM1_ESM.docx]

**SUPPLEMENTARY INFORMATION**

**The interplay between Mn and Fe in *Deinococcus radiodurans* triggers cellular protection during paraquat-induced oxidative stress**

**Sandra P. Santos^1,2^, Yang Yang^3^, Margarida T. G. Rosa^1^, Mafalda A. A. Rodrigues^1^, Claire Bouthier De La Tour^4^, Suzanne Sommer^4^, Miguel Teixeira^1^, Maria A. Carrondo^1^, Peter Cloetens^3^, Isabel A. Abreu^1,^**^*^ **& Célia V. Romão^1,^**^*^

^1^ITQB NOVA, Instituto de Tecnologia Química e Biológica António Xavier, Universidade Nova de Lisboa, Av. da República, 2780-157 Oeiras, Portugal.

^2^*Present address*: iBET, Instituto de Biologia Experimental e Tecnológica, Apartado 12, 2781-901 Oeiras, Portugal

^3^ESRF- The European Synchrotron, CS40220, 38043 Grenoble Cedex 9, France.

^4^Institute for Integrative Biology of the Cell (I2BC), CEA, CNRS, Univ. Paris-Sud, Université Paris-Saclay, 91198 Gif sur Yvette, France.

^*^**Corresponding Authors:**

Célia V. Romão

E-mail: cmromao@itqb.unl.pt

Isabel A. Abreu

E-mail: [abreu@itqb.unl.pt](mailto:abreu@itqb.unl.pt)

**
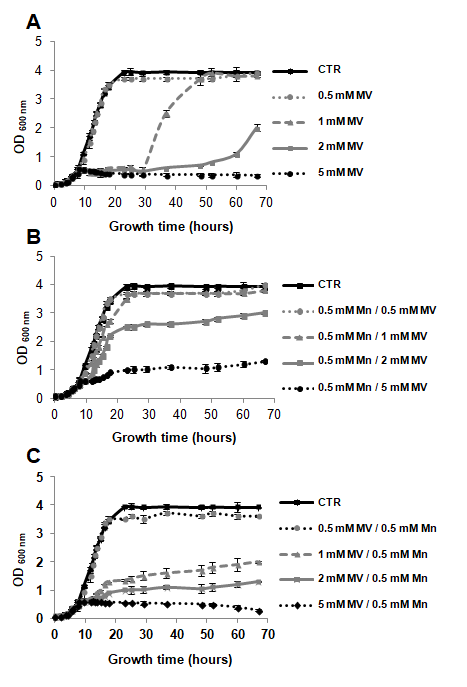
**

**Fig. S1.** ***D. radiodurans* cells submitted to different conditions.** *(A)* Addition of methyl viologen (MV) at different concentrations: 0.5 mM MV, 1 mM MV, 2 mM MV and 5 mM MV. *(B)* Addition of manganese followed by methyl viologen (Mn+MV), the concentrations used were 0.5 mM Mn followed by 0.5 mM MV, 1mM MV, 2 mM MV or 5 mM MV). *(C)* Addition of methyl viologen followed by manganese (MV+Mn), the concentrations used were 0.5 mM MV, 1mM MV, 2 mM MV or 5 mM MV, followed by 0.5 mM Mn. The different compounds were added at OD=0.3, as described in Fig. 1.

**
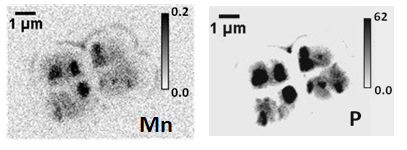
**

**Fig. S2. Localization of elements in *D. radiodurans* wild-type cells under methyl**  **viologen-induced oxidative stress using X-ray fluorescence nano-imaging.** Mn localization (top) and P localization (bottom), the images correspond to the same as presented in Fig. 2A. The images are presented in black and white to have higher contrast, and thus the manganese outside the cells is easily visible.

**
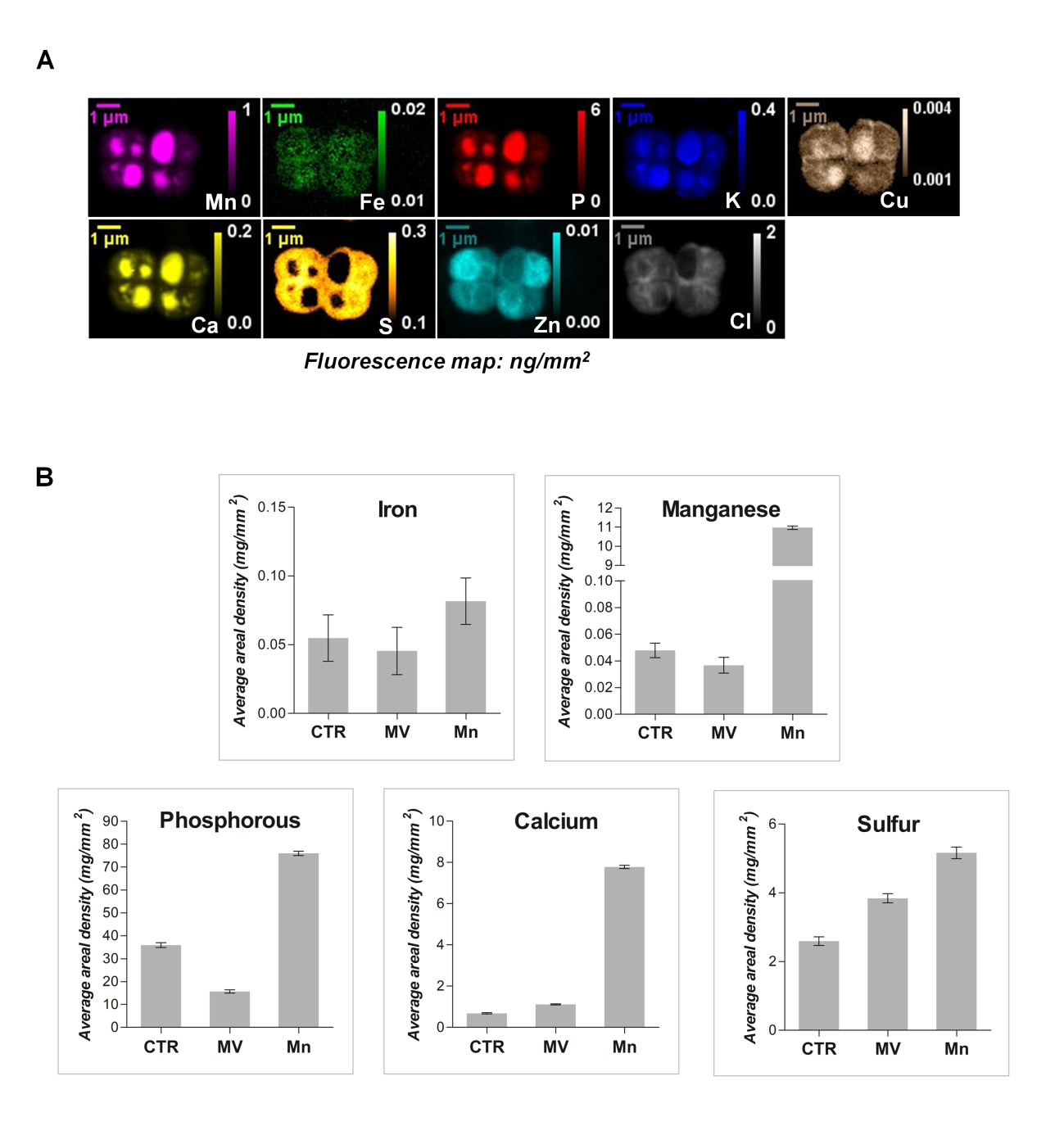
**

**Fig. S3. X-ray fluorescence nano-imaging of elements in *D. radiodurans* wild-type cells collected 2h after adding manganese.** *(A)* Localization of elements in *D. radiodurans* wild-type cells using X-ray fluorescence nano-imaging. *(B)* Average areal densities for individual elements in control (CTR), methyl viologen (MV) and manganese (Mn) conditions. Error bars represent the standard deviations in the image background.

**
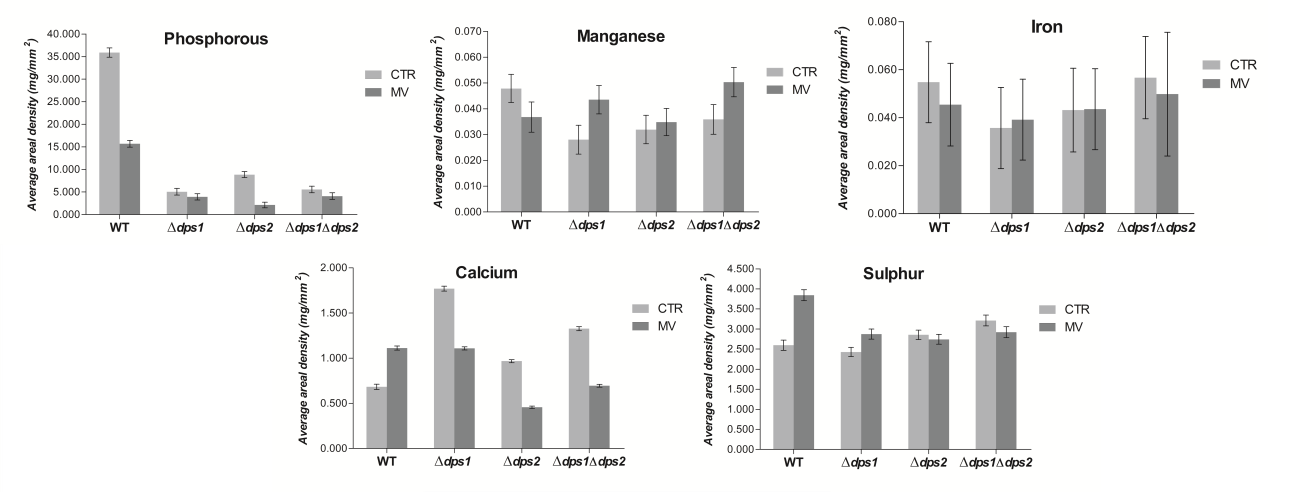
**

**Fig. S4.** **X-ray fluorescence nano-imaging average areal densities for individual elements and species.** The quantification was performed in the *D. radiodurans* WT and in *dps* knockout mutants strains in control (CTR) and oxidative stress (MV) conditions. Error bars represent the standard deviations in the image background.

**
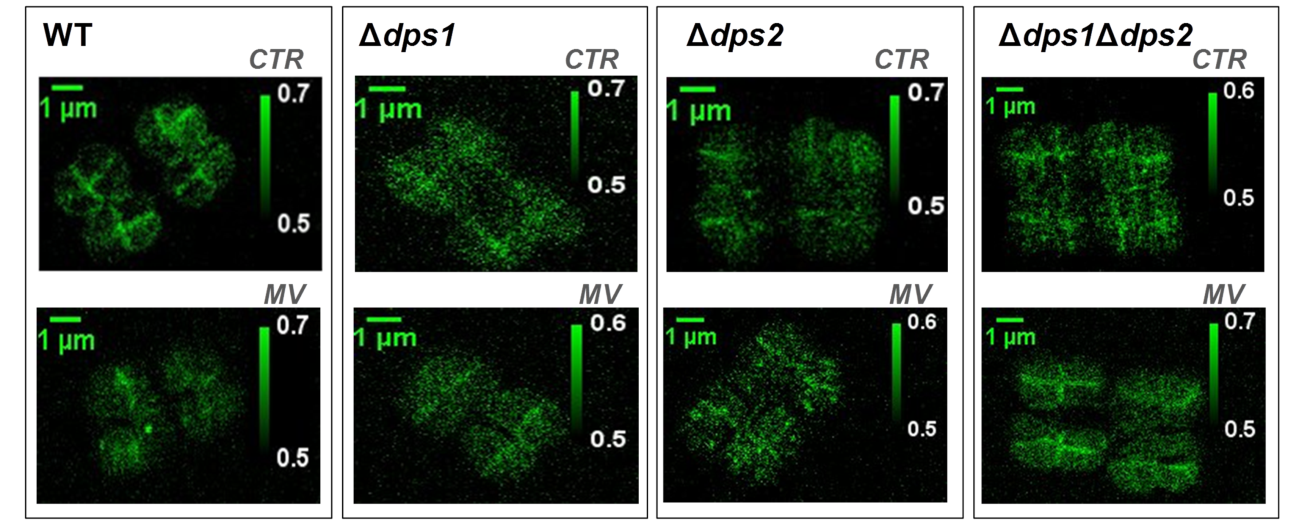
**

**Fig. S5.** **X-ray fluorescence nano-imaging of iron in *D. radiodurans* cells.** Localization of iron using X-ray fluorescence nano-imaging in *D. radiodurans* WT and *dps* knockout mutants strains in control (CTR) and oxidative stress (MV) conditions.

**
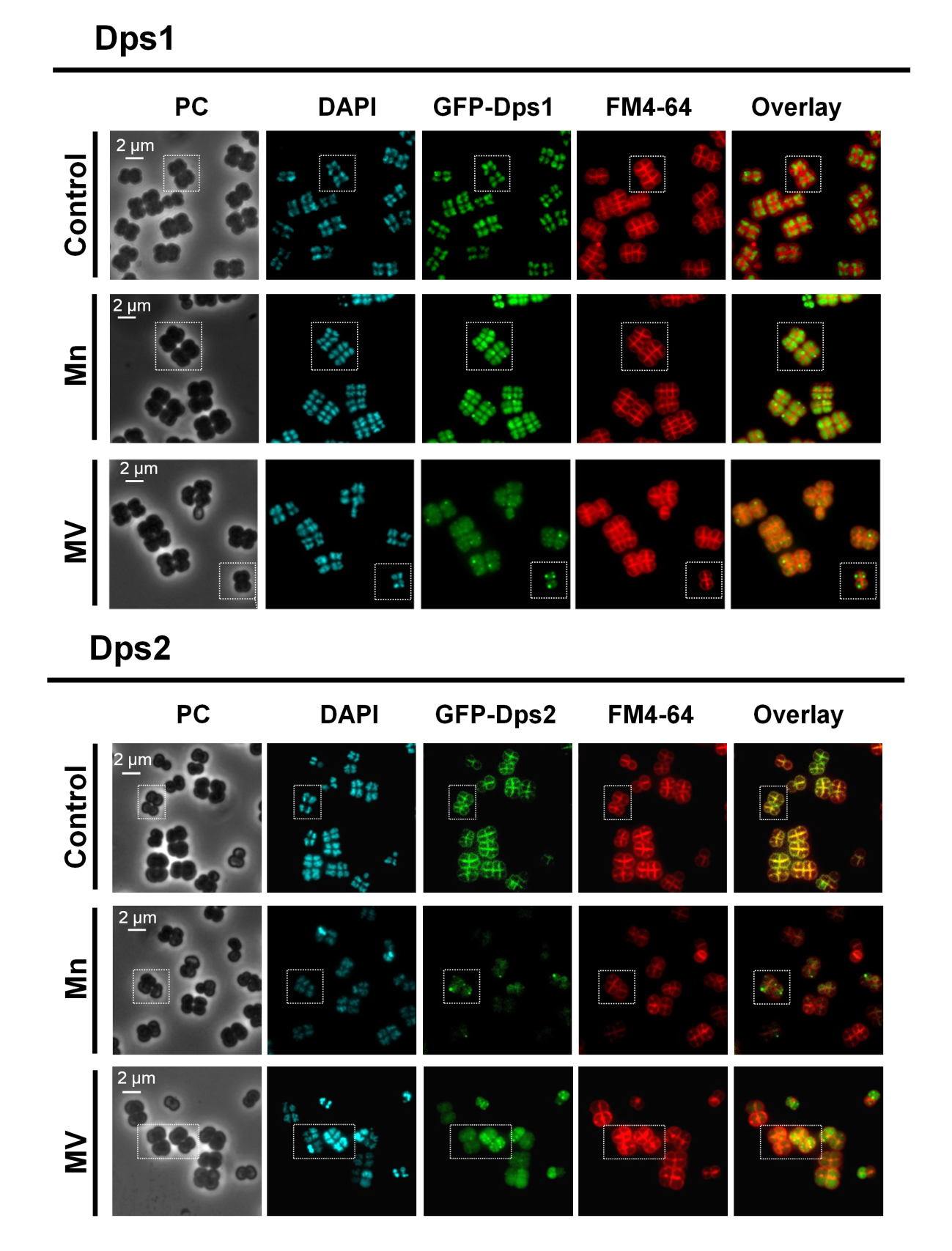
**

**Fig. S6. *D. radiodurans* GFP*-*Dps1 and GFP*-*Dps2 cellular localization using fluorescence microscopy.** The samples analyzed were from control, manganese (Mn) and methyl viologen (MV) conditions at 2 hours. Cell images are represented in phase contrast (PC), stained for DNA using DAPI (blue), fluorescence for GFP*-*Dps constructs (green), and the membranes stained with FM4-64 (red). Overlay corresponds to GFP-Dps with the membrane fluorescence. The region marked by a white square corresponds to the zoom-in represented in Fig. 3.

**
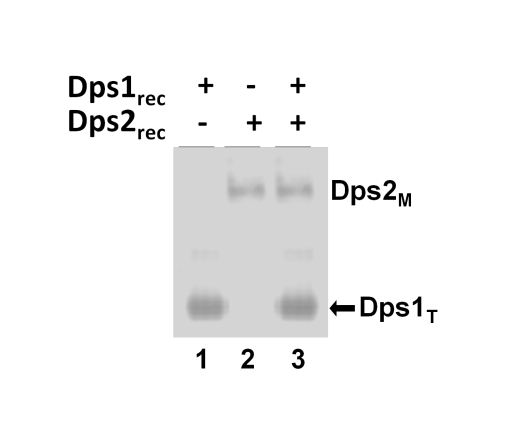
**

**Fig. S7.** **Dps1-Dps2 protein interaction.** Dps1 as trimer (Dps1_T_) (lane 1) and Dps2 full length, Dps2_M_ (lane 2) were used as a control. Dps1 as trimer was incubated with the full-length Dps2_M_ (lane 3). The image correspond to the full-length blot.

**Fig. S8.** **Detection of Dps1 and Dps2 in *D. radiodurans* cellular extracts.** *(A)* Detection of Dps2 in soluble and membrane fractions in *D. radiodurans* wild-type, Δ*dps1* strains and after addition of Dps1 recombinant pure protein (Dps1_rec_) to the cellular extract of Δ*dps1*. Same image as Fig. 4C, but the image corresponds to the full-length blot. *(B)* Detection of Dps1 in *D. radiodurans* Δ*dps2* cellular extracts by Western-blot analysis. The conditions analyzed were: control (CTR), manganese (Mn), methyl viologen (MV) and manganese followed by the addition of methyl viologen (Mn+MV). The time points analyzed were t=0, 15’, 2h and 20h. Bar-charts represent the quantification of the amount of the total Dps1 from four independent samples, using ImageJ software. Dps1_d_ corresponds to the dimeric form of Dps1 and Dps1_T_ to the trimeric form. The image represents the full-length blot.

**
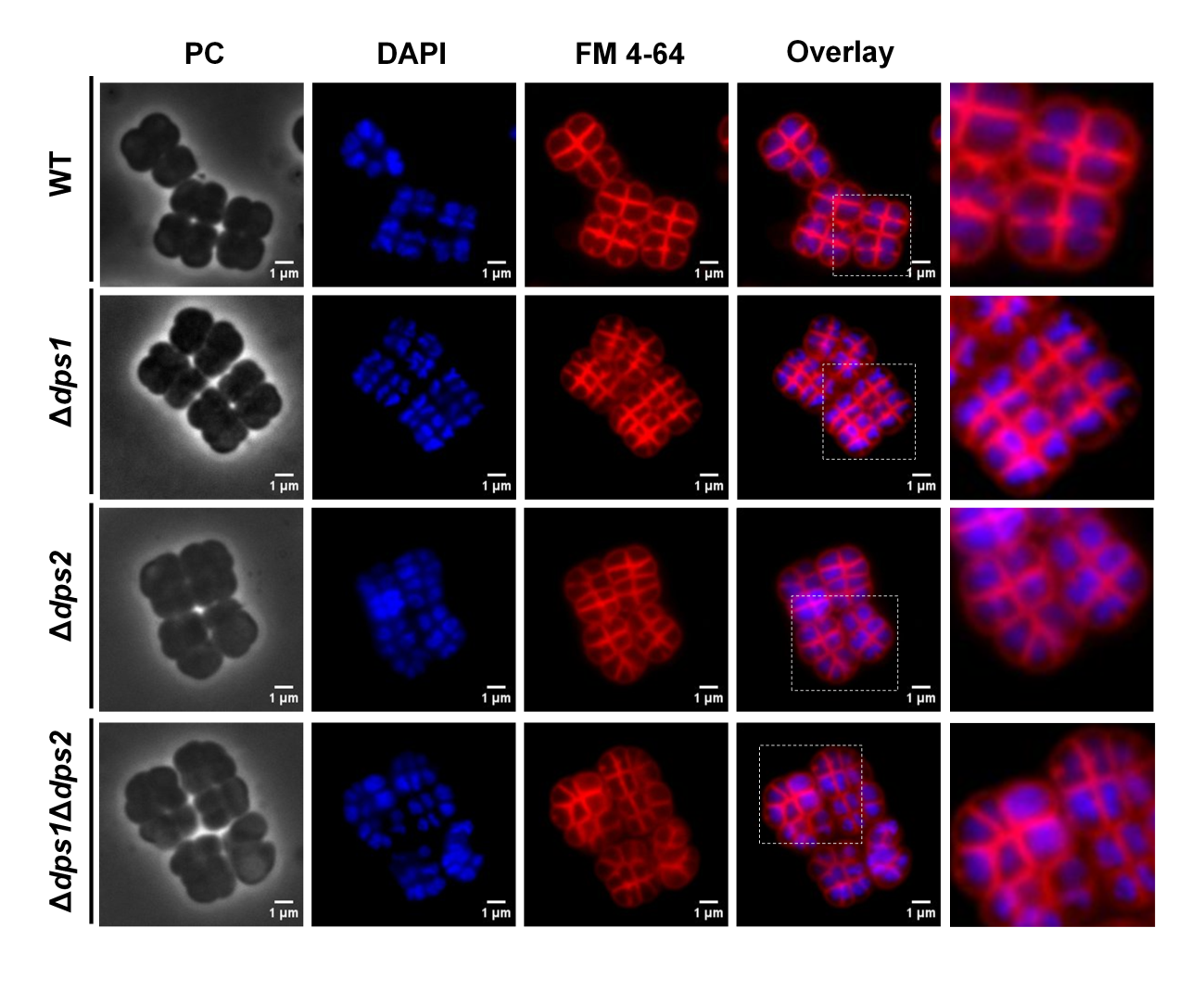
**

**Fig. S9. Fluorescence microscopy of *D. radiodurans* wild-type and *dps* knockout mutants strains in control conditions.** Cells are represented in phase contrast (PC), stained for DNA using DAPI (blue), and the membranes stained with FM4-64 (red). The overlay corresponds to DNA with the membrane fluorescence. The last column corresponds to the zoom area of the white square marked in the overlay images.

**
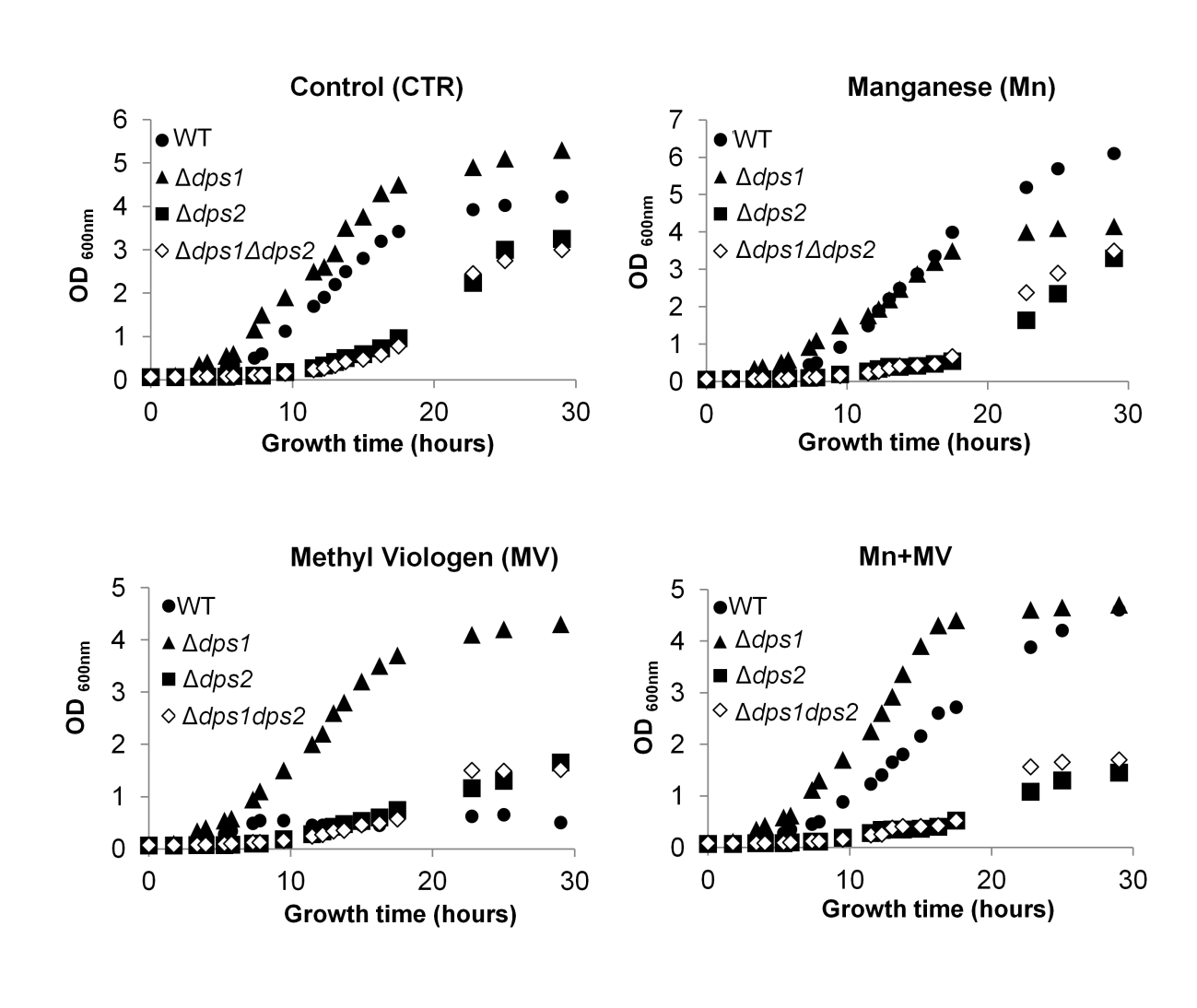
**

**Fig. S10. Growth curves of *D. radiodurans* wild-type cells and *dps* knockout mutants.** Curves representation is according to the type of cell: wild-type (●); Δ*dps1* (▲); Δ*dps2* (■); Δ*dps1*Δ*dps2* (◊) and in the different growth conditions: control (CTR), manganese (Mn), methyl viologen (MV) and manganese followed by methyl viologen (Mn+MV). The data presented is the same as presented in Fig. 1.
